# Supplementary material for: Role of Piezo1 in Terminal Density Reversal of Red Blood Cells
Source: Cells. 2024 Aug 16;13(16):1363. doi: 10.3390/cells13161363 (PMC11352946; doi:10.3390/cells13161363)
Supplement: Supplementary file 1 [file cells-13-01363-s001.zip › cells-3140083-supplementary.pdf]

Supplementary Materials:
Materials & Methods

Red blood cell density separation

Isotonic Percoll density gradient was used to fractionate RBCs according to their density and enrich fractions with young, mature, or senescent RBCs. 90% isotonic Percoll solution (GE Healthcare, Little Chalfont, Buckinghamshire, UK; density 1.13 g/mL) was diluted with a plasma-like medium ((mM): 140 NaCl, 4 KCl, 0.75 $MgSO_4$, 10 glucose, 0.015 $ZnCl_2$, 0.2 alanine, 0.2 glutamate-Na, 0.2 glycine, 0.1 arginine-HCl, 0.6 glutamine, 20 HEPES-imidazole, pH 7.4 at RT). The plasma-like medium was supplemented with 0.1% bovine serum albumin and prepared for each blood sample [15]. In addition, when necessary, Percoll density fractionation was performed with or without Ca^{2+} (1.8-2.0 mM). Microcentrifuge tubes were filled with 13mL of isotonic Percoll, topped with 1mL of blood, and centrifuged at 20,000g for 30 minutes at 35°C (Sorvall RC 5C plus, rotor SM-24). Low (L), medium (M), and high (H) density fractions were collected and were washed three times (2000g, 5 min, room temperature) and resuspended in the plasma-like medium for further characterization.

Hydrolytic activity of PMCA

RBCs PMCA hydrolytic activity was assayed as described by Zemlyanskikh et al. [16] with several modifications. Aliquots of washed RBCs (final hematocrit 10-15%) were added to the medium of the following composition: 50mM Tris-HCl (pH 7.4), 80mM NaCl, 15mM KCl, 3mM $MgCl_2$, 1mM EGTA supplemented with 0.02% Saponin. The cells were incubated for 15-20 minutes on ice. The mixture was transferred to 37°C initially for 5 minutes and $CaCl_2$ was supplemented to reach the final free Ca^{2+} concentration of 10μM. Free Ca^{2+} levels were fixed to the desired concentration by mixing EGTA and $CaCl_2$ in proportions calculated using the following calculator:https://somapp.ucdmc.ucdavis.edu/pharmacology/bers/maxchelator/CaEGTA-TS.htm

To initiate the hydrolytic PMCA activity ATP (3mM final concentration) (Sigma, Catalog number: A2383) was added and the reaction was allowed to occur for 10 minutes and then stopped by the addition of the ice-cold 10% TCA. The mixture was centrifuged, and the released Pi was determined according to Dey et al. [17].

Flow cytometry

The following parameters were detected using flow cytometry (Gallios, Beckman Coulter): the geometric mean of forward (FS) and side (SS) scatter, immature reticulocyte counts (CD71 staining), abundance of PS on the outer membrane (Annexin V staining), RBC membrane surface area for band 3 (eosin 5-maleimide, EMA staining), $[Ca^{2+}]_i$ (Fluo-4 AM), and $[Na^+]_i$ (CoroNa™ Green-AM).

Triple staining was performed with CD71 (Anti Human CD71, eBioscience; Clone: OKT9, Ref: 17-0719-42), Annexin V (Invitrogen; ebiosciences Annexin V apoptosis detection kit, Catalog number: 00-6993-50), and Fluo-4 AM (Invitrogen,2 μM) to distinguish between the young (CD71+) and senescent cells (Annexin V+) and their $[Ca^{2+}]_i$ content. Fluorescent stainings were done in plasma-like medium with 1μL of blood or with Percoll-separated L-fraction cells according to van

Cromvoirt et. al. [14] using Gallios flow cytometer (Beckman Coulter, Indianapolis, USA). $[Na^+]_i$ was measured by utilizing the dye CoroNa™ Green-AM by following the protocol of Iamshanova et al [18] as well as Negulescu et al [19] with substantial modifications as follows: (i) the dye loading was performed at 37°C for 45-60 minutes; (ii) for calibration of CoroNa Green-AM, fluorescence intensity was recorded for the clamped cells with fixed intracellular Na^+ concentrations in plasma-like buffer. To study the effects of different modulators on $[Na^+]_i$, the same experimental paradigm was used as above to perform flow cytometry: (i) Piezo channel was activated by Yoda1 (1.5μM, 30 minutes incubation period) or blocked by GsMTx-4 (2.5μM, 30 minutes incubation period); (ii) Na^+, K^+-ATPase (NKA) and PMCA activity was blocked with Ouabain (100 μM for 45 minutes) as well as with Na-orthovanadate (1mM for 30 minutes) at room temperature (RT). Additionally, in flow cytometry, we examine the capacity of the cells to swell in response to hypo-osmotic stress [20].

Supplementary figures:

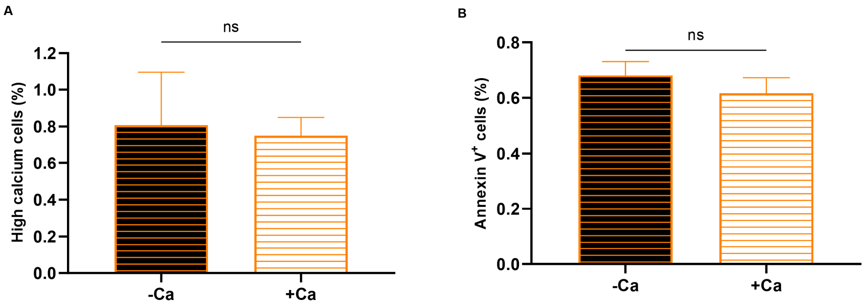

Figure S1; (A) Number of Annexin V+ & (B) Number of "High Ca^{2+}" in the L-fraction after Percoll density gradient separation without or with 1.8-2.0μM Ca^{2+} in the medium (n = 4). Data are presented as mean± SD. ns= not significant.

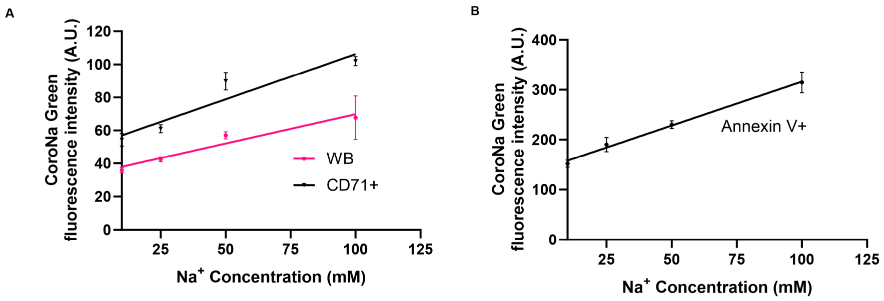

Figure S2; (A,B) Calibration curve for CoroNa Green-AM in unfractionated RBCs (WB), in CD71+ and in Annexin V+ RBCs. The real-time extracellular Na^+ concentrations (0–100 mM) are indicated.

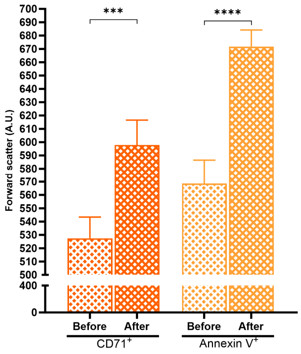

Figure S3; Forward scatter analysis before and after the hypo-osmotic stress test measured in the flow cytometer (n = 5). Data are presented as mean ± SD. ***p<0.001 ****p<0.0001
